# Performance Analysis of Positioning Solution Using Low-Cost Single-Frequency U-Blox Receiver Based on Baseline Length Constraint

**DOI:** 10.3390/s19194352

**Published:** 2019-10-08

**Authors:** Liguo Lu, Liye Ma, Tangting Wu, Xiaoyong Chen

**Affiliations:** 1 Faculty of Geomatics, East China University of Technology, Nanchang 330013, China; lglu@ecit.cn (L.L.); ttwu@whu.edu.cn (T.W.); 2 Xi’an Research Institute of Surveying and Mapping, Xi’an 710054, China; 3 State Key Laboratory of Geo-information Engineering, Xi’an 710054, China; 4 GNSS Research Center, Wuhan University, Wuhan 430079, China; 2012301610164@whu.edu.cn

**Keywords:** baseline length constraint, u-blox receiver, ambiguity resolution, positioning accuracy

## Abstract

With the rapid development of the satellite navigation industry, low-cost and high-precision Global Navigation Satellite System (GNSS) positioning has recently become a research hotspot. The traditional application of GNSS may be further extended thanks to the low cost of measuring instruments, but effective methods are also desperately needed due to the low quality of the data obtained using these instruments. Thus, in this paper, we propose the analysis and evaluation of the ambiguity fixed-rate and positioning accuracy of single-frequency Global Positioning System (GPS) and BeiDou Navigation Satellite System (BDS) data, collected from a low-cost u-blox receiver, based on the Constrained LAMBDA (CLAMBDA) method with a baseline length constraint, instead of the classical LAMBDA method. Three sets of experiments in different observation environments, including two sets of static short-baseline experiments and a set of dynamic vehicle experiments, are adopted in this paper. The experiment results show that, compared to classical LAMBDA method, the CLAMBDA method can significantly improve the success rate of the GNSS ambiguity resolution. When the ambiguity is fixed correctly, the baseline solution accuracy reaches 0.5 and 1 cm in a static scenario, and 1 and 2 cm on a dynamic platform.

## 1. Introduction

The need for Global Navigation Satellite System (GNSS) navigation and positioning is increasing in various fields. However, its application may be limited by its high cost. Compared with expensive measurement receivers, low-cost receivers have many advantages, including low power consumption, small size, portability, and high integration [1,2]. Thus, the low-cost GNSS devices will keep having a wide use in various walks of life. Take the GNSS chipsets, for example, which are now installed in almost every mobile phone, tablet, computer, and other intelligent terminals, are successfully used in position-based services such as pedestrian navigation, vehicle tracking, and social networking [3,4,5,6]. Many studies have been carried out on the data quality assessment and positioning performance of various intelligent terminals, e.g., Google Nexus9, Xiaomi8, HUAWEI Mate20, and the results show that they can generally achieve a positioning accuracy of meter level, or even sub-meter level [7,8,9,10,11].

Besides the intelligent terminals, the receiver developed by the Swiss u-blox company, which could achieve higher precision, is one of the most widely used low-cost receivers. The low-cost u-blox receivers are widely used in aerial vehicle applications [12], mapping surveys [13], geodetic monitoring [14,15], etc., as an alternative to expensive measurement receivers. Different aspects of the positioning performance of this receiver have been evaluated. Stempfhuber [16] studied u-blox data using RTKLIB, and the results showed that this scheme had a positioning accuracy at the centimeter scale. Nevertheless, it was often unstable in practical applications. Rapiński [17] evaluated the observation quality and positioning accuracy of u-blox LEA-6T receiver, and showed that the positioning performance of low-cost receiver is limited by the accuracy of receiver clock. Odolinski et al. [18,19,20,21] experimentally found that the u-blox receiver is in the same order of magnitude as the measurement-type dual-frequency Global Positioning System (GPS)receiver in terms of the ambiguity resolution and positioning performance. Mongredien et al. [12] achieved a short-baseline centimeter-level positioning accuracy using a u-blox receiver and differential correction information. Zuo et al. [22] used a u-blox receiver and measurement antenna to reach a static 1.2 cm and dynamic 2.4 cm positioning accuracy.

In order to further improve the ambiguity, fixed success rate, and baseline solution performance, baseline length information is usually added as a constraint [23,24,25,26]. Among them, Teunissen [26] proposed the CLAMBDA (Constrained LAMBDA) algorithm by merging baseline length information into the ambiguity resolution process. The results showed that it could theoretically obtain a more rigorous fixed solution and significantly improve the success rate of the single-frequency, single-epoch GPS ambiguity resolution [27,28,29]. Therefore, this paper analyzes the low-cost single-frequency GPS and BeiDou Navigation Satellite System (BDS) u-blox receiver data using the classical LAMBDA method and the CLAMBDA method, with a baseline length constraint.

## 2. Methods

In principle, the GNSS baseline models can be cast in the following frame of linear observation equations:

(1) $$\begin{array}{l} E(y)=Aa+Bb,D(y)=Q_{yy},a\in Z^{n},b\in R^{p}\\ E(l)=\left\|b\right\|,D(l)=\delta_{l}^{2} \end{array}$$

where y is the observation vector, a and b are the ambiguity parameter vectors and baseline component parameter vectors, respectively. A and B are the corresponding coefficient matrices, and E(·) and D(·) denote the expectation and dispersion operators, respectively.

According to the least square criterion, the objective function of Equation (1) is expressed as [26]

(2) $$\underset{a\in Z^{n},b\in R^{3},\;\left\|b\right\|=l}{\min}\left\{{\left\|\hat{a}-a\right\|}_{Q_{\hat{a}\hat{a}}}^{2}+\min\left({\left\|\hat{b}(a)-b\right\|}_{Q_{\hat{b}(a)\hat{b}(a)}}^{2}+\sigma_{l}^{-2}{\left(l-\|b\|\right)}^{2}\right)\right\}$$

where $\hat{a}$ and $Q_{\hat{a}\hat{a}}$ are the floating-point solution of the ambiguity and its variance-covariance matrix, respectively, $\hat{b}(a)$ and $Q_{\hat{b}(a)\hat{b}(a)}$ are the conditional expectation and covariance matrix of the baseline components, respectively, a is the integer solution of the ambiguity, and b is the fixed baseline solution.

It can be seen, from Equation (1), that the minimum integer ambiguity vector, satisfying the sum of the ambiguity quadratic form and the baseline component quadratic form, is enumerated in a certain space, that is, the optimal ambiguity vector fixed solution. In this case, the optimal ambiguity fixed solution $\overset{⌣}{a}$ and the corresponding baseline fixed solution $\overset{⌣}{b}$ are satisfied:

(3a) $$\overset{⌣}{a}=\arg\underset{a\in Z^{n}}{\min}\left\{{\left\|\hat{a}-a\right\|}_{Q_{\hat{a}\hat{a}}}^{2}+\underset{b\in R^{3},\;\left\|b\right\|=l}{\min}\left({\left\|\hat{b}(a)-b\right\|}_{Q_{\hat{b}(a)\hat{b}(a)}}^{2}+\sigma_{l}^{-2}{\left(l-\|b\|\right)}^{2}\right)\right\}$$

(3b) $$\overset{⌣}{b}(\overset{⌣}{a})=\arg\underset{b\in R^{3},\;\left\|b\right\|=l}{\min}\left({\left\|\hat{b}(a)-b\right\|}_{Q_{\hat{b}(a)\hat{b}(a)}}^{2}+\sigma_{l}^{-2}{\left(l-\|b\|\right)}^{2}\right).$$

According to Equation (3a), CLAMBDA adds baseline length information to the ambiguity search and baseline fixation process. By expanding the search range of the ambiguity quadratic form, it can find the minimum ambiguity vector satisfying the mixed quadratic form. Considering that the detailed steps for calculating baseline components are not given in the literature [26,27], the calculation process of Equation (3b) is briefly described below.

The baseline component solution satisfies the following mathematical model:

(4) $$\begin{array}{l} E[\hat{b}(a)]=b,D\left(\hat{b}(a)\right)=Q_{\hat{b}(a)\hat{b}(a)}\\ E(l)=\left\|b\right\|,D(l)=\sigma_{l}^{2} \end{array}.$$

Given that Equation (4) is a nonlinear least squares problem, it needs to linearize ‖b‖.

(5) $$\left\|b(a)\right\|=\left\|b(0)\right\|+\frac{x(0)}{\left\|b(0)\right\|}\Delta x+\frac{y(0)}{\left\|b(0)\right\|}\Delta y+\frac{z(0)}{\left\|b(0)\right\|}\Delta z$$

where $b(0)={\left[x(0),y(0),z(0)\right]}^{T}$.

Therefore, its error equation is described as

(6a) $$V_{1}=\Delta b-[\hat{b}(a)-b(0)]$$

(6b) $$V_{2}=\left[\frac{x(0)}{\left\|b(0)\right\|},\frac{y(0)}{\left\|b(0)\right\|},\frac{z(0)}{\left\|b(0)\right\|}\right]\Delta b-[l-\left\|b(0)\right\|]$$

where $\Delta b={\left[\Delta x,\Delta y,\Delta z\right]}^{T}$.

The least square adjustment of Equation (6) is carried out to obtain b(1)

(7) b(1)=b(0)+Δb.

The above iterative steps are repeated, until the difference between b(n) and b(n−1) is within a certain range. In order to speed up the convergence of the least squares, the initial value is selected as follows:

(8) $$b(0)=\hat{b}(a).$$

## 3. Results

In order to effectively analyze the positioning performance of the low-cost single-frequency u-blox receiver under the baseline length constraint, this paper adopted the high-precision GNSS positioning, velocity, and attitude measurement software, KinPOS v2.0 (which can process GPS+BDS single/multi-frequency observations), developed by our research group. Then, two sets of static short-baseline measured data and a set of dynamic on-board test data (the reference station and rover station are both installed in the car with a fixed baseline length) from different observation environments, were selected for comparative analysis with the ambiguity fixing rate and baseline resolution accuracy calculated successively. The basic information concerning the experimental data is shown in Table 1, and the processing configuration is shown in Table 2. The U-blox-M8P receivers, which could only receive single-frequency observations were used in the experiment, and due to the limited observation channels of this receiver, the dual-system data of GPS/BDS were used. In order to better evaluate the performance of LAMBDA and CLAMBDA method, where the latter one takes advantage of the prior baseline length information in the ambiguity search procedure, a single-epoch geometry-based double-difference RTK technique was adopted. The residual ionosphere and troposphere were corrected by the model, the cut-off angle was set as 10°, and the weight of the code/phase was set as 1:100.

It should be noted that, for the static data, the real values of the baseline positions were calculated by the commercial software, CHC Geomatics Office (CGO), developed by CHCNAV, China. For the kinematic data, the real values were calculated by the commercial software, GrafMov, developed by NovAtel, Canada. Moreover, only the fixed solution of GrafMov (Quality number = 1) was adopted. As the true ambiguity values were unknown, the ambiguity-fixed baseline positions were compared to determine whether the ambiguities had been fixed correctly.

### 3.1. Static Data 1

**Figure 1:**
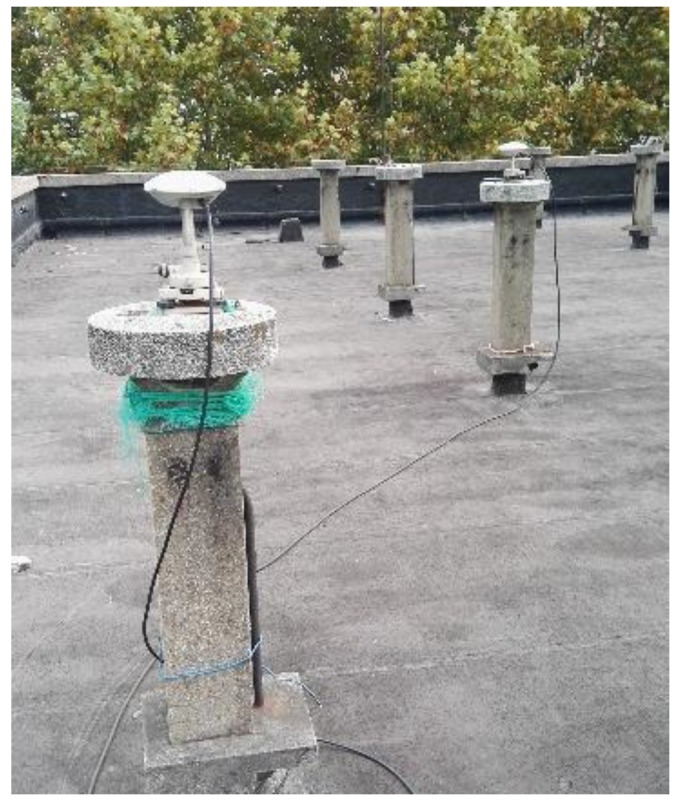
Experimental scene of the static data.

**Figure 2:**
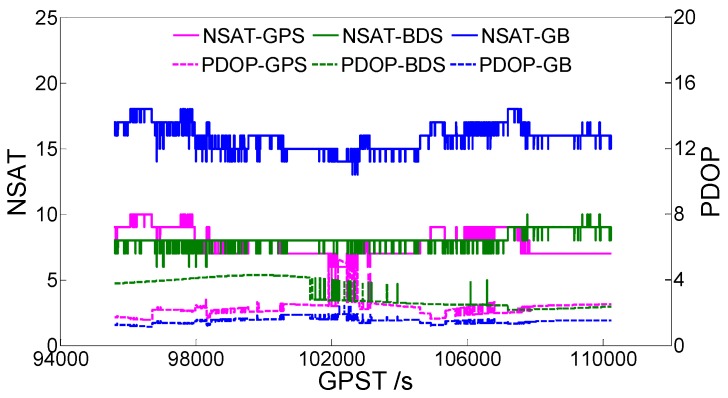
Number of satellites (NSATs) and the position dilution of precision (PDOP) value. GB denotes GPS + BDS.

**Figure 3:**
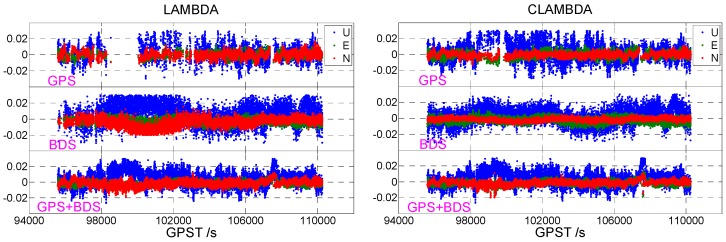
East (E)/north (N)/ up (U) deviation sequence diagram ((**left**) LAMBDA, (**right**) CLAMBDA).

Figure 1 shows the static data 1 acquisition scenario. Figure 2 is a time series of satellite numbers and the position dilution of precision (PDOP) values. Table 3 displays the ambiguity fixed-rate statistics and baseline component root mean square (RMS) statistics in the east (E), north (N), and up (U) directions. Figure 3 exhibits a sequence diagram, showing the deviation of the E/N/U components from the reference value of the LAMBDA and CLAMBDA solutions.

As shown in Table 3, GPS and BDS represent the single GPS and BDS solution, respectively, and GPS+BDS denotes a combination of the GPS and BDS solutions. The correct fixed rate refers to the ratio of epoch numbers with an ambiguity that has been fixed correctly, i.e., the deviation between the fixed baseline component and the reference value is less than 2/2/4 cm in the E/N/U directions, respectively. It can be seen that for the single-frequency single-system solution, due to the small number of satellites, poor satellite geometry, and limited observable values, the fixed rate is significantly lower when the ambiguity is fixed by the LAMBDA method, and the ambiguity fixed rate of the single GPS and single BDS is only 39.56% and 69.75%, respectively. In contrast, the ambiguity fixing success rate using the CLAMBDA method is significantly higher, with the single GPS and single BDS reaching 80.71% and 99.22%, respectively. For the single-frequency dual-system solution, the fixed effect of LAMBDA is markedly improved due to the increase of available observations and the enhancement of the satellite geometry. Currently, the fixed rates of LAMBDA and CLAMBDA are 97.46% and 98.66%, respectively. It is thus clear that CLAMBDA is slightly better than LAMBDA. In addition, from the statistical accuracy of the baseline E/N/U components, the accuracy of CLAMBDA and LAMBDA is comparable or even slightly better than that of the LAMBDA method. CLAMBDA can achieve a positioning accuracy of 0.5 cm, in the horizontal direction, and 1 cm in the elevation.

The same conclusion can be drawn from Figure 3. The number of epochs fixed by CLAMBDA is significantly higher than that of LAMBDA, and the accuracy of the corresponding baseline component is higher. The results of the static u-blox data show that the use of baseline length information to assist the ambiguity search can increase the intensity of the ambiguity resolution model and improve the accuracy and reliability of the ambiguity-fixed solution, thus enhancing the positioning performance.

### 3.2. Static Data 2

**Figure 4:**
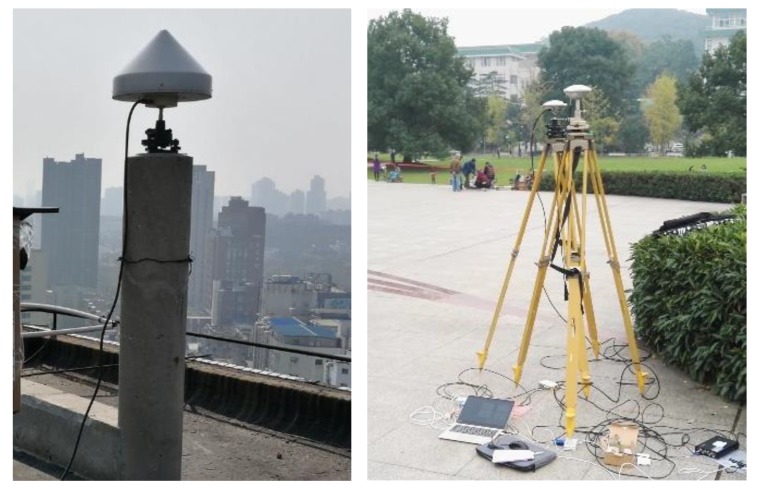
Data acquisition scenario ((**left**) reference station, (**right**) rover station).

**Figure 5:**
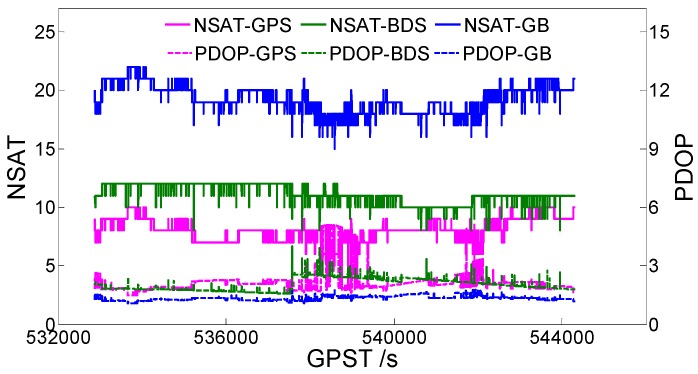
Number of satellites (NSATs) and the PDOP value. GB denotes GPS + BDS.

**Figure 6:**
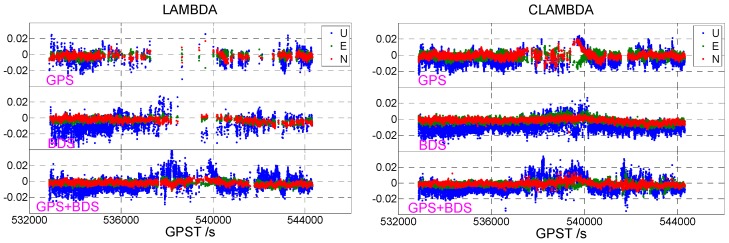
E/N/U deviation sequence diagram ((**left**) LAMBDA, (**right**) CLAMBDA).

Next, we apply the baseline length to static data 2. The static data acquisition scenarios are illustrated in Figure 4 (the rover station is selected from the one on the right picture), and the corresponding common-view satellite number and PDOP value sequence are shown in Figure 5. The statistic results concerning the ambiguity fixed-rate and its baseline component accuracy in E/N/U are shown in Table 4. The deviation sequence diagrams, showing the truth-value and E/N/U components obtained by LAMBDA or CLAMBDA, are drawn in Figure 6.

Similar conclusions can be drawn from Table 4: For both the single GPS or single BDS, the correct ambiguity fixing rate obtained using the CLAMBDA method is significantly higher than that obtained using LAMBDA method. In the case of two systems, the CLAMBDA method also has better performance, and its fixed success rate is increased from 77.69% to 93.91%. As can be seen, from Figure 6, the accuracy of the baseline E/N/U components, obtained using the CLAMBDA method, is slightly higher than that obtained using the LAMBDA method, which is consistent with the statistical results, shown in Table 4. It can clearly be concluded that CLAMBDA can achieve a positioning accuracy of 0.5 cm in the level and 1 cm in the elevation.

### 3.3. Vehicle Data

**Figure 7:**
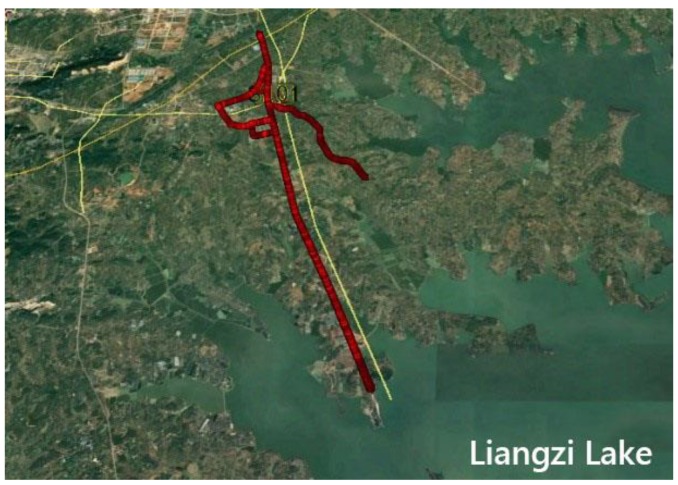
Experimental scene of the dynamic vehicle data.

**Figure 8:**
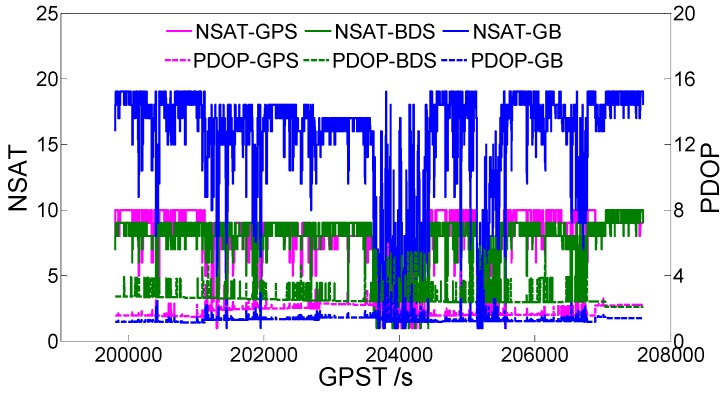
Number of satellites (NSATs) and the PDOP value. GB denotes GPS + BDS.

**Figure 9:**
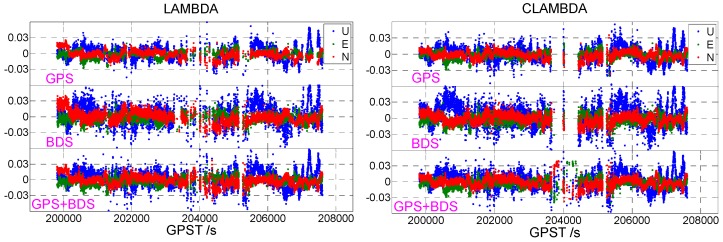
E/N/U deviation sequence diagram ((**left**) LAMBDA, (**right**) CLAMBDA).

Next, we discuss the dynamic scenario. Figure 7 exhibits the vehicle-borne data acquisition scenario. Figure 8 is a time series of satellite numbers and PDOP values. Table 4 indicates the statistical ambiguity fixed rate and the accuracy of the baseline E/N/U components. Figure 9 demonstrates a sequence diagram of the deviation of the E/N/U components, derived from the LAMBDA or CLAMBDA solutions, from the reference value.

For the dynamic experimental data, a conclusion similar to that drawn from the static experiments can be drawn from Table 5. When the u-blox single-frequency single-system data are fixed by the CLAMBDA method, the correct fixed rate is significantly better than that obtained using the LAMBDA method. At the same time, it is also slightly improved in the single-frequency dual-system. From the statistical accuracy of the baseline E/N/U components, the dynamic observation environment is obviously worse than that of the static environment. This is because the dynamic observation environment is often complex and changeable, and the phenomena of the abnormal observation values and satellite occlusion occur more frequently. It can be seen from the statistical results, that the baseline component solutions based on the CLAMBDA and LAMBDA methods are nearly equivalent, and the positioning accuracy can reach 1 cm in the level and 2 cm in the elevation. Near GPST 204000s, there is a difference between the results obtained using CLAMBDA and those obtained using LAMBDA, which is caused by the occlusion of buildings. An abnormality can also be seen in the number of satellites and the PDOP value, shown in Figure 8. In other time shots, the baseline component deviation sequence diagram corresponding to CLAMBDA is more intensive, that is, CLAMBDA has a higher fixed success rate.

## 4. Conclusions

In this study, two sets of static data and a set of vehicle-dynamic low-cost u-blox measured data were used to analyze the positioning performance of the LAMBDA and CLAMBDA ambiguity resolution methods under a baseline length constraint. Considering the two aspects of the ambiguity fixed success rate and baseline solution accuracy, the following three conclusions can be drawn from the experimental analysis:

(1) Without baseline length constraints, BDS single-frequency data can obtain a higher ambiguity fixed success rate than GPS single-frequency data. GPS+BDS dual-system single-frequency data can significantly improve the accuracy of the ambiguity resolution.
(2) Under a baseline length constraint, the CLAMBDA method can greatly improve the fixed success rate of ambiguity for single-frequency single-system data (for instance, the single GPS and BDS increased by 41.15% and 29.47% in static data 1, respectively, and the dynamic single GPS and BDS increased by 19.39% and 19.96%, respectively). Considering the single-frequency dual-system data, the CLAMBDA method has a relatively greater accuracy due to the high fixed success rate of the LAMBDA method itself.
(3) Given that the ambiguity is fixed correctly, CLAMBDA and LAMBDA have the same positioning accuracy and both can reach the centimeter level. Under a static observation environment, the positioning accuracy can reach 0.5 cm in the horizontal direction and 1 cm in the vertical direction, while in the dynamic case, the positioning accuracy can reach 1 cm in the horizontal level and 2 cm in the vertical direction.

## Figures and Tables

**Table 1:** Data information.

Data	Scene	Time Span	Receiver	Antenna	Baseline Length
1	Static	4 h	U-blox-M8P	Trimble Zephyr Model 2	3.28 m
2	Static	3:10 h	U-blox-M8P	UniStrong UA91 3D/Trimble Zephyr Model 2	1637.41 m
3	Vehicle	2:10 h	U-blox-M8P	Trimble Zephyr Model 2	1.13 m

**Table 2:** Basic processing configuration.

System and Frequency	GPS (L1) and BDS (B1)
Process Model	Single-epoch RTK
Ambiguity Resolution	LAMBDA/CLAMBDA
Sampling Interval	1 s
Cut-off Angle	10°
Ionosphere Model	Klobuchar
Troposphere Model	Saastamoinen
Weight of Code and Phase	1:100

**Table 3:** Positioning precision statistics.

Method	Satellite System	RMS-E/m	RMS-N/m	RMS-U/m	Fixed Success Rate
LAMBDA	GPS	0.0036	0.0044	0.0094	39.56%
	BDS	0.0040	0.0066	0.0127	69.75%
	GPS + BDS	0.0031	0.0039	0.0093	97.46%
CLAMBDA	GPS	0.0038	0.0029	0.0101	80.71%
	BDS	0.0040	0.0025	0.0092	99.22%
	GPS + BDS	0.0032	0.0031	0.0089	98.66%

**Table 4:** Positioning precision statistics.

Method	Satellite System	RMS-E/m	RMS-N/m	RMS-U/m	Fixed Success Rate
LAMBDA	GPS	0.0033	0.0034	0.0086	20.44%
	BDS	0.0035	0.0036	0.0121	31.71%
	GPS + BDS	0.0030	0.0030	0.0088	77.69%
CLAMBDA	GPS	0.0032	0.0036	0.0085	70.66%
	BDS	0.0036	0.0028	0.0109	93.58%
	G + B	0.0029	0.0028	0.0088	93.91%

**Table 5:** Positioning precision statistics.

Method	Satellite System	RMS-E/m	RMS-N/m	RMS-U/m	Fixed Success Rate
LAMBDA	GPS	0.0062	0.0078	0.0127	72.59%
	BDS	0.0077	0.0107	0.0189	73.44%
	G + B	0.0069	0.0087	0.0152	98.03%
CLAMBDA	GPS	0.0063	0.0071	0.0133	91.98%
	BDS	0.0085	0.0085	0.0200	93.40%
	G + B	0.0076	0.0084	0.0152	98.06%
